# Food insecurity as a cause of adiposity: evolutionary and mechanistic hypotheses

**DOI:** 10.1098/rstb.2022.0228

**Published:** 2023-10-23

**Authors:** Melissa Bateson, Gillian V. Pepper

**Affiliations:** ^1^ Centre for Healther Lives and Biosciences Institute, Newcastle University, Newcastle upon Tyne, NE2 4HH, UK; ^2^ Department of Psychology, Northumbria University, Newcastle upon Tyne, NE1 8ST, UK

**Keywords:** food insecurity, obesity, insurance hypothesis, energy balance, metabolic rate, metabolic adaptation

## Abstract

Food insecurity (FI) is associated with obesity among women in high-income countries. This seemingly paradoxical association can be explained by the insurance hypothesis, which states that humans possess evolved mechanisms that increase fat storage to buffer against energy shortfall when access to food is unpredictable. The evolutionary logic underlying the insurance hypothesis is well established and experiments on animals confirm that exposure to unpredictable food causes weight gain, but the mechanisms involved are less clear. Drawing on data from humans and other vertebrates, we review a suite of behavioural and physiological mechanisms that could increase fat storage under FI. FI causes short-term hyperphagia, but evidence that it is associated with increased total energy intake is lacking. Experiments on animals suggest that unpredictable food causes increases in retained metabolizable energy and reductions in energy expenditure sufficient to fuel weight gain in the absence of increased food intake. Reducing energy expenditure by diverting energy from somatic maintenance into fat stores should improve short-term survival under FI, but the trade-offs potentially include increased disease risk and accelerated ageing. We conclude that exposure to FI plausibly causes increased adiposity, poor health and shorter lifespan.

This article is part of a discussion meeting issue ‘Causes of obesity: theories, conjectures and evidence (Part II)’.

## 1. The food insecurity-obesity paradox

**Figure 1.**
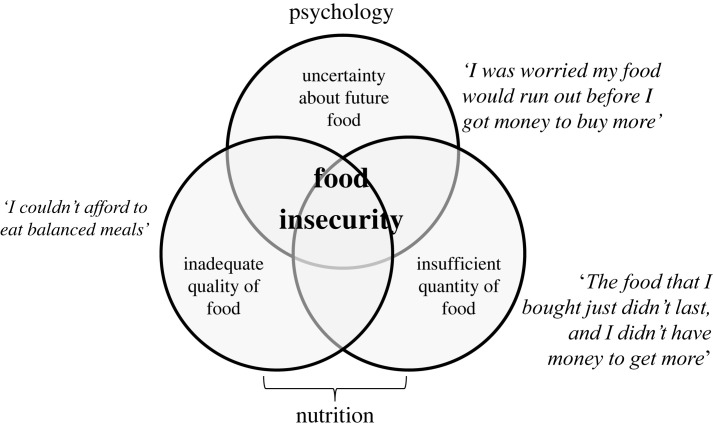
Food insecurity (FI) is a construct that comprises a psychological domain related to uncertainty about future access to food and nutritional domains related to periodic variability in the quantity and nutritional quality of food consumed. The statements in italics are examples of items probing each domain taken from the United States Department of Agriculture Adult Food Security Survey Module, one of the standard instruments used to measure FI.

In a 1995 case report entitled ‘*Does hunger cause obesity?*’ William Dietz describes an obese American girl from a poor family dependent on welfare. The family frequently lacked enough money to buy healthy food and resorted to cheap, energy-dense foods to prevent hunger [1]. The situation faced by this family is commonly referred to as food insecurity (henceforth, FI), a construct that captures periodic experience of insufficient quantity and quality of food and anxiety about future food scarcity, but not chronic energy deficit [2,3]. FI is measured with questionnaires that probe the presence and severity of the different domains of the construct over a defined period (usually the last 12 months; figure 1). These instruments yield a continuous scale [4,5], that in practice is often used to categorize respondents as food secure or insecure. Many studies have confirmed positive associations between FI and obesity, establishing the so-called ‘food insecurity-obesity paradox’ as a robust phenomenon [6–11]. A meta-analysis of this literature estimates the overall odds of overweight and obesity as 21% higher for food-insecure individuals, with larger effects in adult females and high-income countries, resulting in the odds of overweight and obesity being about 50% higher for food-insecure adult women in high-income countries [12]. While this effect is only moderate in size, for comparison, it is larger than the increased odds of high body weight associated with carrying a risk allele of the *FTO* gene [13]. FI therefore deserves attention as a potential environmental cause of variation in human adiposity. Our aim in this article is to review evolutionary and mechanistic hypotheses explaining the association between FI and obesity observed in high-income countries.

Biologists make a distinction between proximate and ultimate explanations for physiological and behavioural responses [14,15]. Ultimate explanations are concerned with why a response evolved, whereas proximate explanations are concerned with the mechanisms underlying the response within an individual. Dietz wrote, ‘At least two possibilities could explain the association of hunger and obesity in the same patient … the increased fat content of food eaten to prevent hunger … represents the most likely reason for the association of obesity and hunger. An alternative possibility is that obesity may represent an adaptive response to episodic food insufficiency’ [1]. Dietz’ first explanation is a proximate mechanism: food-insecure individuals gain weight because they switch to consuming higher-energy density foods; whereas his second is an ultimate explanation: food-insecure individuals gain weight because it is adaptive to have greater fat stores as insurance against energy shortfall. Although Dietz presents these explanations as alternatives, ultimate explanations always require underlying proximate mechanisms, meaning that proximate and ultimate explanations can be complementary [14]. Thus, while switching to energy-dense foods could be a non-adaptive constraint of poverty (because they are cheaper), it could also be the output of an evolved psychological mechanism that has been positively selected because it delivers increased adiposity as an adaptive response to periodic food insufficiency [16].

Dietz’ adaptive explanation for the association between FI and obesity captures the logic underlying the insurance hypothesis, an ultimate-level explanation for FI-induced weight gain that has its origins in evolutionary ecology [12]. The insurance hypothesis states that humans, in common with other vertebrates, possess evolved mechanisms that increase fat storage as insurance against energy shortfall when access to food is unpredictable. In §2, we briefly review the theoretical assumptions underlying this hypothesis. A key prediction is that unpredictable food should cause increased fat reserves. However, while the insurance hypothesis posits the existence of proximate-level mechanisms that deliver fat gain under FI, it does not specify what these are; the positive energy balance necessary to produce weight gain could arise from changes in either energy intake or expenditure. In §3, we review experiments exploring the effects of unpredictable food on body weight, food consumption and energy expenditure in humans and animals, and in §4, we review epidemiological evidence on the associations between FI, diet and energy expenditure in humans. We conclude that increased energy intake is unlikely to be the primary cause of FI-induced weight gain. In §5, we discuss the suite of behavioural and physiological mechanisms triggered by FI that could deliver a positive energy balance. Finally, in §6, we consider the trade-offs required to increase fat storage in the absence of increased energy intake.

## 2. The insurance hypothesis

Although in his original 1995 paper Dietz speculated about ‘an adaptive response to episodic food insufficiency’ ([1], see also [17]), the insurance hypothesis was not formalized for humans until 2017, when the social science literature on FI was integrated with a theoretically grounded literature on body-weight regulation in birds originating in the 1980s [12]. The insurance hypothesis is based on the assumption that fat provides a buffer against energy shortfall during periods when food is not available [18]. However, carrying fat has costs as well as benefits [19]; increased fat increases energy requirements [20], reduces locomotor performance [21] and increases the risks of injuries resulting from having a heavier body [22]. In humans, increased fat also has social costs arising from stigma and discrimination [23]. It follows that the optimal amount of fat depends on the unpredictability of food: the greater the probability of energy shortfall, the higher the optimal fat stores [12]. Thus, the set point (or zone) around which body weight is regulated should be higher under FI.

**Figure 2.**
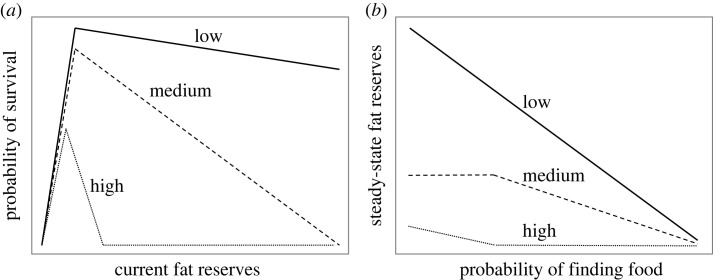
(*a*) Three functions describing assumed theoretical relationships between current fat reserves and probability of survival that differ in the costs of carrying additional fat reserves (low, medium or high costs). (*b*) Corresponding functions describing how optimal steady-state fat reserves are predicted to decrease as the probability of finding food increases and thus food insecurity (FI) decreases given the assumptions in panel (*a*). When the cost of additional fat is low, increasing FI is predicted to be associated with higher optimal fat reserves and increased fat reserves as FI increases. Whereas when the cost of additional fat is high, increasing FI is predicted to be associated with lower optimal fat reserves and little change in fat reserves as FI increases. We speculate that the low-cost scenario could model the situation in women and the high-cost scenario the situation in men. Redrawn from [12].

The insurance hypothesis arose from formal optimality models based on principles of Darwinian fitness maximization created to explain body-weight variation in birds facing unpredictable access to food [24–28]. These models were couched in terms of starvation-predation trade-offs, but there is nothing in the logic that restricts their applicability to birds, or that requires the cost of fat to be increased predation risk [19]. The central assumption is the existence of an asymmetrical fitness function relating probability of survival (or reproduction) to fat reserves, whereby probability of survival (or reproduction) declines rapidly below a threshold of reserves, owing to starvation (or failure of reproduction), but declines more slowly above this threshold, owing to the increasing costs of higher body weight (figure 2*a*). A fitness function of this basic shape is biologically plausible, and is supported in humans by the positively skewed distribution of body mass index, which shows that very high body fat can be compatible with survival [29]. The specific shape of the function will depend on the biology of the species and is likely to vary with age, sex and current reproductive state in humans [30]. For example, the threshold is predicted to be at a higher level of reserves in adult women than men, because women require more fat to fuel reproduction. The shape of this function determines both the amount of fat an individual should carry when access to food is secure and also, critically, how fat reserves should respond to changes in the unpredictability of food. Results from altering the shape of the function in a formal model show that increasing the costs of additional body fat reduces the amount of fat it is optimal to store in response to unpredictable food (figure 2*a*,*b*). If the costs of additional fat are higher in males than females, we speculate that this could explain the sex difference in the response to FI observed in humans, but further work is needed to understand this sex difference (see [12] for further discussion).

It follows from the above theory that humans and other vertebrates should possess evolved mechanisms that respond to cues of actual or potential food scarcity by delivering adaptive regulation of body fat. While the insurance hypothesis assumes that these mechanisms evolved because they were adaptive in the evolutionary environment, it is not necessarily the case that the response to FI seen in high-income countries today is adaptive. Evolved mechanisms can deliver maladaptive responses if there is an evolutionary mismatch, whereby the mechanisms are operating in an environment that is different from that in which they originally evolved [31]. Mechanisms that detect FI could misfire if modern humans are constantly bombarded with cues of anticipated food scarcity via the media [16]. Additionally, mechanisms that increased fat storage adaptively in the past may overshoot in the modern food environment owing to changes in the energy density or nutrient composition of available food. Thus, while the insurance hypothesis provides an ultimate explanation for why food-insecure humans are fatter than food-secure humans, it does not entail that the absolute levels of obesity observed in high-income countries maximize survival [29].

## 3. Does food insecurity cause weight gain?

A key prediction of the insurance hypothesis is that exposure to FI should cause an increase in adiposity and hence body weight (figure 2*b*). The observed association between FI and obesity in humans is compatible with the insurance hypothesis, but does not prove causation, because both variables could be caused by a third variable, most obviously poverty [32–34]. Longitudinal studies measuring changes in weight in response to changes in FI are proposed as a stronger test of causality (e.g. [35]), but they do not eliminate third-variable explanations. Moreover, the requirement in longitudinal studies to measure FI at least twice, doubles the measurement error for a variable that is already likely to be measured with poor precision, resulting in low power to detect true effects of changes in FI on weight [36]. Longitudinal studies reporting non-significant associations between FI and weight gain therefore do not provide strong evidence against FI causing weight gain [35]. Ultimately, only randomized controlled experiments that manipulate FI and measure changes in weight can prove that FI causes weight gain.

### (a) Experiments in humans

Ideally, participants would be randomly assigned to conditions in which FI is imposed for long enough for detectable changes in body weight. However, manipulating FI experimentally is not straightforward. FI comprises nutritional and psychological domains (figure 1) and it is currently unknown which of these are necessary or sufficient to trigger weight gain. There have been no attempts to experimentally manipulate all domains of FI simultaneously in human participants.

An alternative approach is to manipulate a single domain of FI. In one such study, a psychological domain of FI referred to as anticipated food scarcity [37], was manipulated by requiring participants to watch a video depicting either climate change-induced food shortages, or a control video not related to food. Immediately following the video, participants' preferences for different foods were measured. In three such experiments, viewing the video that increased anticipated food scarcity caused increased preference for more energy-dense foods in female participants [16]. These results are presented as supporting the insurance hypothesis, based on the assumption that increased preference for energy-dense foods could lead to increased energy intake and hence weight gain. This result suggests that in the absence of any changes in nutrition, uncertainty about future food availability alone is sufficient to cause immediate behavioural changes that could fuel weight gain. Further research is required to establish how long these changes last and whether they actually translate into weight gain.

Although there are no studies on humans explicitly designed to manipulate the nutritional domains of FI, there is a series of potentially relevant experiments that manipulated variability in the number of meals eaten daily [38–41]. Variability in meal number was manipulated by assigning participants for two weeks to either a regular condition, in which they ate six meals daily at fixed times, or an irregular condition, in which they ate a mean of six meals daily, but the number varied between three and nine meals on any day. Total energy intake over the two-week intervention was equal in the two conditions, and in two studies, participants were provided with all of their meals, ensuring that diet quality and total energy intake were identical [39,40]. Although not designed as a manipulation of FI, the variability in daily energy intake generated by the irregular condition can be seen as mimicking the fluctuations in food availability characteristic of FI (see §4a). Since participants in both conditions were informed about the number and timing of their meals, uncertainty about future access to food was not manipulated in these experiments, only variability. The irregular condition caused a decrease in dietary thermogenesis. Although this did not cause significant weight gain over the two weeks of the intervention, it would do, all else being equal, if sustained for longer. Thus, in the absence of increased total energy intake, variability in the temporal pattern of intake is sufficient to cause weight gain.

### (b) Experiments in non-human animals

Starting in the 1980s, coupled with the theoretical developments underpinning the insurance hypothesis, there is a substantial empirical literature exploring the effects of unpredictable food on body-weight regulation in birds [12]. Laboratory experiments on various bird species provide compelling evidence that unpredictability in energy intake causes increases in body fat and body weight. Unpredictability in energy intake is most commonly manipulated by periodically completely removing food or reducing the quantity of food available. For example, in a 19 week experiment, European starlings were allocated to either an unpredictable condition, in which otherwise unlimited food was removed for a randomly chosen 5 h period on five days each week, or a control, in which unlimited food was always available [42]. In common with the human experiments described above, this manipulation generated variation in the pattern of eating. Birds in the unpredictable condition gained body weight (*ca* 2.7% in week 19) and fat relative to the controls, but although they could theoretically compensate for the periods of deprivation by eating more when food was available, they actually ate slightly less in total. Simultaneously with gaining weight, the birds in the unpredictable condition showed accelerated erythrocyte telomere shortening and reduced feather regrowth relative to the controls, suggesting that increased fat deposition was fuelled by reduced investment in somatic maintenance, indicative of reduced basal energy expenditure.

A variation on the above method for generating variability in access to food, is to remove food for multiple shorter time periods within each day, creating within-day unpredictability in the pattern of eating. In three longitudinal experiments with starlings, birds spent one week in a baseline secure condition, in which food was unlimited, before being switched to an insecure condition, in which food was removed for 12 out of 20 randomly chosen 20 min periods each day ([43] experiments 1–3). Another variation on the same approach is to deliver food on an operant schedule, whereby subjects are trained to peck a key to obtain a small food reward, and reduce the probability that a peck results in food delivery to induce short-term unpredictability in access to food. In a longitudinal experiment, starlings spent one week in a baseline secure condition, in which a key peck always yielded 10 s of food access, before being switched to an insecure condition, in which the probability of a key peck yielding food was reduced ([43] experiment 4). This operant method has the advantage of providing detailed data on foraging motivation [44]. Across all four of these experiments, the birds gained weight in the insecure conditions (*ca* 3% increase) relative to the secure baseline and this difference occurred within a week of experiencing unpredictable access to food [43]. Birds in the operant experiment responded to the insecure condition by increasing the number of pecks they performed each day and consuming more during their periods of food access, but these changes in behaviour were insufficient to compensate for the reduction in probability of accessing food. Thus overall, the birds gained weight in the insecure conditions despite consuming less food each day (*ca* 13% reduction in the mass of food consumed). In two of the experiments ([43] experiments 1 and 3), measurements were made of the energy density of guano, and this declined slightly in the insecure condition (*ca* 1% decrease), suggesting that insecure birds were absorbing more metabolizable energy from their food. There was also evidence from one of the experiments ([43] experiment 3) that the birds spent more time engaged in inactive roosting behaviour, a possible mechanism for reducing energy expenditure. No direct measures of energy expenditure were made in these experiments, but the increase in metabolizable energy retained was too small to compensate for the decrease in food intake, suggesting that the insecure birds must have reduced their energy expenditure to fuel weight gain.

Experiments in several species of birds, including great tits, greenfinches, tufted titmice and black-capped chickadees, have confirmed that manipulating unpredictability in access to food can cause weight gain [45–48], but not all experiments in birds report an effect [49–52]. For example, while most experiments on starlings show that unpredictable food causes weight gain [42,43,53–58], zebra finches do not show this effect [50,52]. Some of this variation might reflect species differences in the value of carrying fat reserves [46], but there are currently too few directly comparable studies in most species to be sure whether the differences observed are robust. It is likely that some variation between experiments in the response to unpredictable food is explained by the severity of the manipulation: starlings subjected to an unpredictable 40% reduction in feeding time each day lost weight [59], whereas birds subjected to an unpredictable 29% reduction gained weight [53]. In species such as the starling that have been extensively studied, it is clear that individual state moderates the size of the effect. Unpredictable food causes larger weight increases in birds that are lighter at baseline [43,58], in birds that face greater conspecific competition for food [43] and in birds that are currently photosensitive [57]. Interestingly, given the sex difference in humans, there is no evidence that male and female starlings differ in their response to unpredictable food [42]. We speculate that costs of fat may be more similar in male and female starlings than in humans, because starlings are less sexually dimorphic than humans in both morphology and behaviour.

The majority of experiments reviewed above were designed to test the predictions of the optimality models cited in §2 and were conducted in non-domesticated bird species. Passerine species, such as the starling, are excellent models for studying the behavioural ecology of FI. From a life-history perspective, starlings are similar to humans in being a long-lived (20+ years), invasive species capable of adapting to different environments. Starlings regulate their fat reserves rapidly in response to changes in environmental conditions, making them ideal for experimental studies. It is worth emphasizing that the weight increases reported in starlings are modest (*ca* 3%) and are not a model of obesity. The importance of these studies is in demonstrating that the set point or zone around which body weight is regulated can be altered by the food environment.

With growing interest in understanding the physiological impacts of FI, mammalian models are required, and animal models of FI in rats and mice are starting to appear [60–62]. In a recent experiment, female Sprague-Dawley rats were assigned for 12 weeks to either an insecure condition, in which there was unpredictable variation in the number and size of meals a day, or a control, in which, the number and size of daily meals was constant and energy intake was fixed to be identical to the mean intake in the insecure condition ([61] experiment 2). Following this manipulation, the rats were all placed on unlimited food and the previously insecure rats gained more weight than the controls. Individual food consumption was not measured during the unlimited phase, but weight gain was attributed to a combination of hyperphagia and a reduction in resting energy expenditure evidenced by slower weight loss in the insecure rats during a 24 h period of food deprivation. In this experiment, all rats were maintained on mild caloric restriction during the intervention phase to ensure all food was consumed and mean intake was equalized, which might explain why weight gain was not observed in the insecure rats until they were placed on unlimited food. As we argued above for the starling experiments, whether weight gain occurs during unpredictable feeding is likely to depend on the degree to which animals are calorically restricted during the intervention.

In all of the animal studies described thus far, diet quality was held constant, demonstrating that shifts in macronutrient intake under FI are not necessary for weight gain. However, the above study in rats additionally explored the interaction between unpredictable feeding and a high-fat/high-sugar diet. In rats fed a high-fat/high-sugar diet, unpredictability caused increased motivation to earn sucrose pellets, a lasting increase in meal size and greater weight gain during an unlimited feeding phase, relative to rats fed regular chow ([61] experiment 1), showing that poor diet quality can exacerbate the effects of unpredictable food on adiposity.

In summary, experimental manipulations of FI in humans still need to be developed and direct evidence that FI causes weight gain in humans is therefore lacking. Animal experiments demonstrate conclusively that unpredictable food can cause weight gain, even in the absence of increased total food intake or a change in diet quality. Therefore, the mechanisms responsible for FI-induced weight gain are not restricted to those that increase energy intake and include mechanisms that increase energy retention and decrease energy expenditure.

## 4. Is food insecurity associated with differences in energy balance in humans?

Most of our knowledge of the effects of FI in humans comes from observational studies of participants classified as food secure or insecure using standard questionnaires that reference the period immediately prior to data collection. In this section, we review studies conducted in high-income countries (predominantly North America and the UK) that have used this approach to measure associations between FI and aspects of behaviour, cognition and physiology relevant to energy balance and hence weight gain.

Any account of FI-induced weight gain must explain why energy intake exceeds energy expenditure [63]. It is commonly assumed that the primary explanation for the association between FI and obesity is increased food consumption and hence energy intake [1,64,65]. However, reduced energy expenditure has been proposed as an additional or alternative explanation for FI-induced weight gain in humans [17,42,43,61,66]. In the original statement of the insurance hypothesis, the focus was on decision mechanisms regulating food intake [12], but there is nothing in the logic of the models that restricts the underlying mechanisms to those regulating eating. The models described in §2 could be modified to include either behavioural decisions or physiological responses affecting energy retention or energy expenditure without impacting the predictions. Animals use a range of different behavioural and physiological mechanisms to regulate body weight adaptively under conditions of starvation or overfeeding [18,67], and it is parsimonious to assume that humans do the same. Hence, we review studies related to the effects of FI on both energy intake and energy expenditure.

Two types of observational study on the effects of FI exist. In the first type, which are typically large-scale epidemiological studies, data are self-reported by participants who are living freely in their normal environments and buying their own food (§4a). In the second type, participants classified as either food secure or food insecure in their normal environments are invited into the laboratory for collection of data under standardized conditions (§4b).

### (a) Free-living participants

Studies using 24 h dietary recalls, in which participants report everything they have consumed in one or more 24 h periods, show that the nutritional quality of food-insecure diets is relatively poor. Food-insecure participants have less diverse intake, consuming a smaller number of distinct foods per consumption event [68]. They consume fewer fruits, vegetables and dairy products and have lower intakes of vitamin A, vitamin B6, calcium, magnesium and zinc [69]. In terms of relative macronutrient intake, food-insecure participants have lower intake of fibre and protein and higher intake of carbohydrate [68,70]. They also consume more ultra-processed foods (UPFs) [71]. Despite these differences in diet quality, studies conducted in high-income countries generally show that total daily energy intake either does not differ systematically between food-secure and food-insecure women [2,68,70,72,73], or is lower in food-insecure women [74,75]. The largest effects of FI on diet emerging from these studies are related not to how much is eaten, but to the temporal pattern of eating, with food-insecure adults showing greater temporal variability in their food intake. In a representative American sample, food-insecure participants were also more variable in the time gaps between eating within days. They were also more variable between days in the time at which they first ate and the number of times they ate [68]. Similar results were reported in a sample of British adults, suggesting that these features of food-insecure diets generalize across Western populations [70].

There is some evidence that food-insecure adults are less physically active and that this difference is caused by lower motivation for exercise. For example, in a representative American sample, minutes of physical activity per week were estimated from questionnaires and by accelerometry [76]. FI was associated with failing to meet guidelines for physical activity according to both self-reported and objective measures. In a study of American rural families, FI was associated with lower readiness to encourage and enable physical activity [77]. FI is robustly associated with depression and disordered sleep [78]. Given that core symptoms of depression include fatigue and sometimes psychomotor retardation and increased sleep, we speculate that these effects could contribute to alterations in energy balance under FI.

There is no direct evidence in humans that FI is associated with reduced basal energy expenditure, but FI is associated with shorter leucocyte telomere length and other measures indicating increased biological age [79,80]. These data are compatible with the hypothesis that food-insecure humans invest less energy in somatic maintenance, which is a component of basal energy expenditure, although other interpretations also exist (see §6).

### (b) Laboratory studies

Studies measuring food consumption objectively in the laboratory show that when provided with unlimited food, food-insecure participants consumed more calories than food-secure participants. For example, in a study in which British students were asked to taste a selection of snack foods, women who were food insecure consumed more calories during the test [81]. Similarly, in a study in which American adults stayed in a residential facility for three days where they could eat freely from vending machines filled with diverse foods, food-insecure participants ate about 700 kcal per day more than food-secure participants and ate a diet relatively higher in carbohydrates and fats [82]. In this latter study, the food-insecure participants also had higher disinhibition, hunger and binge-eating scores assessed via questionnaires. In a follow-up study in which American adults were confined in a respiratory chamber for 24 h and fed an amount of food calculated to meet their energetic needs based on their sex, age and size, previously food-insecure participants reported greater hunger and showed metabolic and hormonal changes associated with higher energy intake [83]. In this study, no effect of previous FI status on total energy expenditure was found [83], but there were limitations because the participants were predominantly male and physical activity was limited.

The laboratory studies therefore report different associations between FI and energy balance from those reported in free-living participants. One possible explanation for this discrepancy is that humans are known to under-report food intake and this bias is larger in those with higher body mass index [84,85], creating doubt over the accuracy of self-reported data on energy intake described in §4a. However, the discrepancy could also reflect a true difference explained by the differing circumstances of the participants. Whereas food-insecure participants were hungrier and chose to eat more when they were presented with unlimited food in the laboratory, in their normal environments they were not always able to eat as much as they wanted owing to the constraints of FI. This account is given some support by several of the animal studies reviewed in §3, where although animals with unpredictable access to food displayed hyperphagia during periods when food was available and gained weight, they ate less in total than controls with unrestricted food [42,43,45].

It is perhaps not surprising that free-living food-insecure participants eat a nutritionally poorer diet in a more temporally variable way, since these are components of the definition of FI (figure 1). However, the associations described in §4a raise the question of which differences arise as constraints of FI and which might be adaptive responses to it predicted by the insurance hypothesis. Temporal variability in access to food is likely to be a constraint arising from lack of money or time, whereas changes in macronutrient intake could occur either as the result of constraints in the food available under FI, or from adaptive shifts in preference to maintain energy balance or promote weight gain. Reductions in the intake of fibre and protein under FI are likely to result from constraints owing to the relatively high costs of fruits, vegetables, meat and dairy. Whereas, the finding that food-insecure participants eat diets higher in carbohydrates and fats when diverse foods are freely available [82] lends support to increased preference for these nutrients under FI (see [16]). Higher disinhibition, hunger and tendency to binge eat are all plausibly adaptive cognitive responses to a food-insecure environment selected to increase energy intake, but they could also be pathological symptoms of dysregulated eating caused by repeated exposure to insufficient food [82,83]. Lower physical activity could be explained either as a constraint arising from lack or time or opportunity for exercise, or as an adaptive shift in motivation and behaviour to save energy. Finally, depression could be a non-adaptive consequence of chronic stress associated with FI, or alternatively, some depressive symptoms such as fatigue and psychomotor retardation could be adaptations to reduce energy expenditure [86].

**Figure 3.**
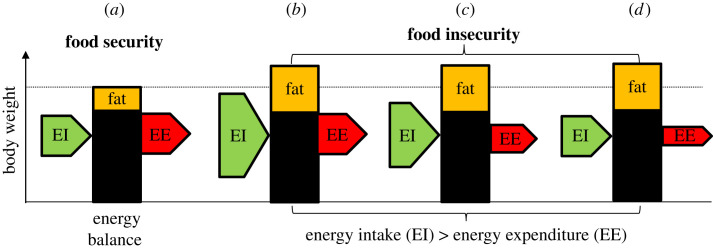
(*a*) In energy balance, energy intake (EI, the green arrow) equals energy expenditure (EE, the red arrow). (*b*)–(*d*) Three scenarios delivering increased body fat and weight that could be triggered by FI: (*b*) increased EI only, (*c*) both increased EI and reduced EE, and (*d*) decreased EE only. Scenario (*a*) is commonly assumed to occur in humans, but experiments in humans and animals suggest that scenarios (c) and (d) can occur in response to variable and unpredictable food.

In summary, while FI is associated with changes in cognition and behaviour that lead to increased energy intake when food is available, it is not clear that increased energy intake is the primary mechanism causing weight gain under FI (figure 3). Reduced energy expenditure on both physical activity and resting metabolic rate could also contribute to FI-induced weight gain. While these changes in energy balance could arise as a consequence of constraints imposed by the environment and dysregulated control of energy balance, they could also represent a suite of changes in response to FI that underpin the adaptive regulation of body weight predicted by the insurance hypothesis.

## 5. Proximate explanations for food insecurity-induced weight gain

The insurance hypothesis predicts that optimal fat stores should be higher under FI and therefore, that the set point or zone around which body weight is regulated should be higher. By contrast, non-adaptive hypotheses for FI-induced weight gain rely on positively biased errors in body weight regulation. Irrespective of whether the changes in body weight are adaptive or non-adaptive, the proximate mechanisms responsible for FI-induced weight gain in humans are poorly understood [43,61,73,87]. In this section we review hypotheses for why FI triggers or facilitates a positive energy balance, many of which depend on the same mechanisms proposed to explain obesity more generally. We organize this section around known features of FI that are either components of its definition (figure 1) or that emerged as correlates of FI in §4 and consider how each feature might cause a positive energy balance.

### (a) Stress

Chronic stress is commonly cited as a proximate mechanism driving FI-induced weight gain [8,73,88]. Anxiety over access to sufficient food is a defining feature of FI and the intermittent hunger caused by FI can result in increased psychological stress [78,89]. At the physiological level, there is evidence that FI is associated with chronically elevated cortisol levels [90,91]. In humans, chronic psychological stress and elevated cortisol levels are associated with increased appetite for fats and sugars, increased energy intake and adiposity [92–94], and experimentally elevated glucocorticoid levels cause increased energy intake [95].

In order to provide insurance, increased fat stores need to precede actual food scarcity. Therefore, under the insurance hypothesis humans should have evolved the capacity to anticipate future food scarcity. We therefore predict that humans should respond to information predicting future food scarcity and that a physiological stress response could be part of the mechanism involved here. Supporting this prediction, anticipated food scarcity is sufficient to trigger an acute increase in preference for high-energy density foods [16]. This response occurred in the absence of general changes in affective state, suggesting a specific response to information about food scarcity, although improved controls are needed to confirm this. However, it is not yet known whether anticipated food scarcity alone causes changes in stress hormone levels or adiposity.

### (b) Increased variability and unpredictability in patterns of eating

Data from both humans and animals point to temporal variability in energy intake as a candidate cue of FI sufficient to trigger mechanisms delivering weight gain. In humans, greater within-day variability in the time intervals between eating was the dietary variable most strongly associated with FI [68]. Moreover, this variable partially statistically mediated the association between FI and obesity, supporting a causal role for variability in temporal patterns of eating in weight gain [68]. The strongest evidence that variability in eating patterns causes positive energy balance comes from the experiments described in §3a showing that eating irregular meals for two weeks caused a reduction in dietary thermogenesis in adult women [38–41]. These studies used a powerful cross-over design whereby participants were tested in both regular and irregular meal conditions in a counterbalanced order, thereby eliminating between-individual differences in metabolic rate that could obscure subtle effects in cross-sectional observational studies (e.g. [83]).

It is currently unclear whether unpredictability in the temporal pattern of eating is necessary to cause weight gain, since no human studies have manipulated unpredictability in the timing of food availability. The optimality models reviewed in §2 predict that unpredictability in access to food should be important, because more insurance is required if the longest periods of deprivation that will be encountered are unknown. Moreover, unpredictability is central to the difference between FI and voluntary intermittent fasting regimes, which are suggested to have opposite effects on body weight [96], but which can both involve long periods without food.

A number of animal experiments have attempted to separate effects owing to variability in food access from effects owing to unpredictability in food access. For example, starlings gained less weight in a predictable-variable condition, in which food was systematically removed for the second 30 min of every hour throughout the day, compared with an unpredictable-variable condition, in which there was a 50% probability of food being removed every 30 min [55]. However, this design confounded unpredictability with longer periods of food deprivation (when 30 min deprivations occurred successively). Although this experiment does not prove a greater effect of unpredictability, it does show that a more variable pattern of food availability within a day is important in triggering weight gain in starlings. In a different design that kept the period of food deprivation constant, starlings gained weight when switched to an unpredictable-variable condition, in which food was removed for a random 4 h period during the day, but the gain was no greater than that seen in a predictable-variable condition, in which food was removed for 4 h at the same time each day [53]. Similarly, starlings provided with a cue predicting the onset of a 5 h period of food deprivation gained a similar amount of weight to birds provided with an uninformative cue [97]. One interpretation of these latter two studies is that variability in access to food is sufficient to trigger weight gain in birds, with no greater effect when variability is also unpredictable. However, this interpretation relies on the birds having learnt the predictability in the predictable-variable conditions during the experiment, because unlike humans, animals cannot be informed of the feeding schedule in advance. In the absence of proof that birds in the predictable-variable conditions knew when food deprivation was scheduled to occur, it is unclear whether unpredictability increases weight gain and further experiments are required to resolve this question.

Although the insurance hypothesis predicts larger effects on body weight when food is unpredictable rather than simply variable, whether this prediction is supported will depend on the nature of the mechanisms that have evolved to detect cues of unpredictability; if variable food sources were typically also unpredictable in the evolutionary environment (which seems quite plausible), then mechanisms could have evolved that do not distinguish between predictable and unpredictable variability. Further experiments in humans and animals comparing the effects of different temporal patterns of food availability and different amounts of information about future food availability are required to understand the role of unpredictable food in weight gain.

The effects of variable and unpredictable food could operate through several different mechanisms. First, unpredictability is likely to be stressful, inducing increases in energy intake through the mechanisms reviewed in §5a. Second, unpredictable food will prevent learning of when meals are expected [98]. This in turn will prevent the acquisition of anticipatory (cephalic) hormonal responses to food that contribute to homeostasis [99]. We speculate that the resulting reduction in control of blood glucose and fats could be a proximate mechanism linking unpredictable eating with adiposity. Finally, more variable food availability involves some longer gaps between eating that will induce greater hunger and consequent hyperphagia when food becomes available (feast-famine cycles). In support of this, FI is associated with faster eating, larger meal sizes and increased total food consumption when humans and animals are given unlimited access to food [43,61,81,82]. Eating fast may interfere with hormonal responses providing feedback on appetite and satiety during eating and is associated with higher body weight in humans [100]. Longer gaps between eating may additionally trigger reduction in metabolic rate as an adaptive response to fasting [18].

### (c) Changes in diet quality

#### (i) Reduced fibre intake

A hypothesis that has received little attention is that FI could trigger an increase in the amount of metabolizable energy retained in the body [43]. In healthy human subjects, only 90% of dietary energy enters the metabolizable energy pool as a result of losses through faeces and urine [101]. Thus, while the energy content of foods consumed is the primary determinant of energy intake, variation in digestion, absorption and excretion of energy could impact energy retention and fibre intake is likely to play a role here. Randomized controlled trials in humans suggest that increased dietary fibre intake reduces body weight and adiposity independently of calorie restriction [102]. The mechanism for this effect could be via the gut microbiome, that is known to be affected by dietary fibre intake and is associated with differences in nutrient absorption [103–105]. Supporting this hypothesis, FI is associated with an altered gut microbiome in humans [106]. We therefore speculate that changes in the gut microbiome resulting from of reduced fibre intake under FI could mediate positive energy balance in the absence of increased energy intake. Alternatively, it is possible that FI could alter gut morphology to increase absorptive area and gut passage time, although we are not aware of any evidence for this in humans. Like many vertebrates, starlings have plastic gut morphology, rapidly increasing gut volume in response to lower-energy density diets [107,108]. Furthermore, unpredictable access to food caused a reduction in the energy density of guano in starlings despite no change in the fibre content of their diet [43], suggesting increased nutrient retention under FI. An increase in metabolizable energy retained under FI could arise either as a non-adaptive side effect of changes in diet forced by FI, or as an adaptive change in food preferences or gut morphology that has evolved to induce a positive energy balance.

#### (ii) Reduced protein intake

A hypothesis not yet considered in the FI literature is that increased energy intake could be a non-adaptive effect of evolved mechanisms that adaptively regulate protein intake. The protein leverage hypothesis assumes that animals, including humans, prioritize defence of a protein intake target [109]. Given the observation that food-insecure diets are relatively low in protein, the protein leverage hypothesis predicts that total food and hence energy intake will passively increase in order to achieve the protein target. This hypothesis is attractive because it offers a testable mechanistic explanation for FI-induced weight gain arising from established differences in macronutrient intake. Protein leverage and the insurance hypothesis are non-mutually exclusive explanations and could contribute independently to FI-induced weight gain.

#### (iii) Increased carbohydrate intake

Under the carbohydrate-insulin model, increased intake of refined carbohydrates with a high glycaemic load, is implicated in increased fat storage and weight gain [110,111]. According to this model increasing adiposity causes increased energy intake to compensate for the loss of available energy stored in fat. Increased carbohydrate intake is a possible proximate explanation for increased energy intake and body weight under FI.

#### (iv) Increased ultra-processed food intake

UPF intake is associated with higher total energy intake [112] and increased UPF intake caused increased energy intake in a randomized controlled trial [113]. While the mechanism responsible for this effect is unclear, higher UPF intake is another possible proximate explanation for increased energy intake and body weight under FI.

In summary, multiple features of FI are likely to cause increased energy intake via a range of mechanisms, many of which have been extensively studied in the obesity literature. These include: stress, longer periods without eating, unpredictable eating, reduced fibre and protein intake and increased carbohydrate and UPF intake. However, a problem with increased energy intake as the primary mechanism underpinning FI-induced weight gain is that it predicts that FI should be associated with higher total energy intake in free-living participants, but there is currently little evidence for this (§4a). Furthermore, changes in macronutrient intake cannot explain changes in energy balance in experimental studies in humans and animals where diet quality is held constant [39,40,43,61]. Therefore, changes in macronutrient intake are not necessary to explain FI-induced weight gain, although they could exacerbate the obesogenic effects of FI [61]. On the basis of the evidence reviewed in this article, we argue that increased retention of metabolizable energy and reduced energy expenditure are likely to be central to FI-induced weight gain (figure 3*c*). Recent studies have revealed unexplained variation in basal metabolic rate in humans [114,115] and we speculate that FI could be a cause of some of this variation.

## 6. The insurance hypothesis and energetic trade-offs

Increasing fat stores within the body requires energy, and in the absence of sufficient energy intake, trade-offs must occur within the body to fuel adaptive fat storage. Energetic trade-offs have been shaped by natural selection, and when energy is limited, investment in responses critical for short-term survival are prioritized over growth, somatic maintenance, immunity and reproduction. Experimental studies in birds have attempted to measure the trade-offs that fuel rapid fat gain when food is limited and unpredictable. For example, starlings exposed to unpredictable food reduced somatic maintenance (measured by accelerated telomere loss and reduced feather regrowth) [42] and black-capped chickadees downregulated the most costly components of their immune response (measured by lower acute phase protein concentration and lower fever temperature) [48]. There is therefore evidence that birds trade off decreased probability of starvation in the short-term against decreased future health and longevity.

Similar evolved trade-offs could be occurring in food-insecure humans. FI in humans is associated with increased odds of disease and reduced lifespan [88,116–120]. Whether FI and disease are independently caused by poverty or whether FI directly drives the development of disease via reduced investment in somatic maintenance and repair is debated [88]. However, the evidence presented in this review provides a pathway whereby FI could directly cause disease and accelerated ageing as a result of evolved energetic trade-offs necessary to fuel adaptive fat storage.

## 7. Conclusion

FI is robustly associated with obesity, with the largest effects seen in adult women in high-income countries. Based on animal experiments showing that unpredictable food causes increased body fat, we argue that this association is likely to be causal. FI is therefore a candidate cause of variation in adiposity seen in high-income countries.

The mechanisms underlying FI-induced weight gain are currently poorly understood and may include both increases in energy intake and decreases in energy expenditure (figure 3). Reductions in physical activity, dietary thermogenesis and basal energy expenditure could all contribute to reduced energy expenditure under FI. Effects of FI on basal energy expenditure, which constitutes 60–70% of the human energy budget, are currently understudied and need further investigation. Increased retention of metabolizable energy is an additional mechanism that could contribute to positive energy balance under FI that needs to be considered.

**Figure 4.**
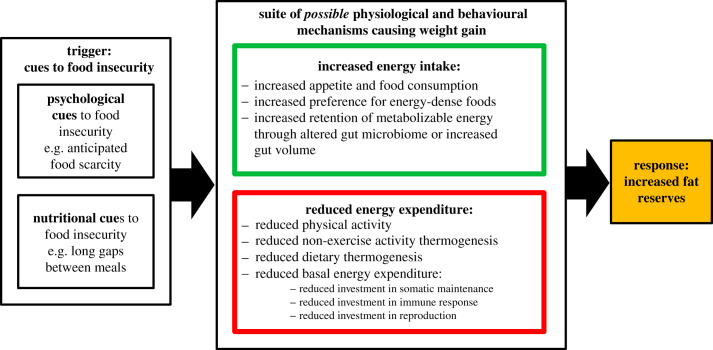
The insurance hypothesis predicts that humans and other vertebrates possess evolved mechanisms that detect cues of food insecurity and regulate energy intake relative to energy expenditure to produce adaptive regulation of fat stores and hence body weight. This figure depicts some of the possible mechanisms that we hypothesize could be involved.

We have presented the insurance hypothesis as an ultimate explanation for FI-induced weight gain. The hypothesis states that FI-induced weight gain is the output of mechanisms that evolved to deliver increased fat stores as insurance against energy shortfall (summarized in figure 4). The insurance hypothesis is unique in predicting that weight gain should be anticipatory and should thus occur in response to cues predicting future food scarcity. The insurance hypothesis leads to the prediction that many of the known correlates of FI, such as changes in diet quality, physical activity and fatigue, may be adaptive responses to FI, selected because they deliver a positive energy balance, rather than constraints imposed by the environment. This change in perspective could have implications for public health interventions designed to mitigate the negative impacts of FI. The strategic allocation of energy to fat that occurs under FI may force evolved trade-offs within the body that could explain why FI is associated with increased odds of developing disease and reduced lifespan. The insurance hypothesis therefore provides a plausible systems-level account of many of the known negative impacts of FI on health and longevity.

## Data Availability

This article has no additional data.
